# Poly(U) RNA-templated synthesis of AppA

**DOI:** 10.1261/rna.052696.115

**Published:** 2015-10

**Authors:** Deepa Puthenvedu, Teresa Janas, Irene Majerfeld, Mali Illangasekare, Michael Yarus

**Affiliations:** 1 Department of Molecular, Cellular and Developmental Biology, University of Colorado, Boulder, Colorado 80309-0347, USA; 2 Department of Biotechnology and Molecular Biology, University of Opole, 45-032 Opole, Poland

**Keywords:** ribodinucleotide, reproduction, cofactor, coenzyme, template

## Abstract

Simple nucleotide templating activities are of interest as potential primordial reactions. Here we describe the acceleration of 5′-5′ AppA synthesis by 3′-5′ poly(U) under normal solution conditions. This reaction is apparently templated via complementary U:A base-pairing, despite the involvement of two different RNA backbones, because poly(U), unlike other polymers, significantly stimulates AppA synthesis. These interactions occur in moderate (K^+^) and (Mg^2+^) and are temperature sensitive, being more efficient at 10°C than at 4°C, but absent at 20°C. The reaction is only slightly pH sensitive, despite potentially relevant substrate p*K*_a_’s. Kinetic data explicitly support production of AppA by interaction of stacked 2MeImpA and pA nucleotides paired with a single molecule of U template. At a lower rate, AppA can also be produced by a chemical reaction between 2MeImpA and pA, without participation of poly(U). Molecular modeling suggests that 5′-5′ joining between stacked or concurrently paired A's can occur without major departures from normal U-A helical coordinates. So, coenzyme-like 5′-5′ purine dinucleotides might be readily synthesized from 3′-5′ RNAs with complementary sequences.

## INTRODUCTION

By many kinds of calculations, RNA and ribozymes are likely to have played early roles in life on Earth (Atkins et al. 2011). However, even small known ribozymes can contain dozens of required ribonucleotides, making them statistically infrequent (Kennedy et al. 2008), unstable because adjacent nucleotides can be aligned for easy hydrolysis (Soukup and Breaker 1999), burdened with replication that is easily poisoned by chirally related sugars (Joyce et al. 1984b), and difficult to extricate from stable double-stranded replicative intermediates (Sievers and Von Kiedrowski 1994; Engelhart et al. 2013).

Many such problems are simultaneously solved if early RNAs are small, perhaps as small as dinucleotides (Yarus 2011). For example, imidazole-activated nucleotides are easily formed from nucleoside 5′ oligophosphates (Lohrmann 1977). Such reactive imidazolides subsequently give rise to 5′-5′ linked dinucleotides (Lohrmann and Orgel 1978). Similar imidazolide-activated dinucleotides, on the basis of published rate data, appear kinetically capable of appearing spontaneously and possibly even replicating (Yarus 2012), even in prebiotic pools that get random, infrequent, varying supplies of ribonucleotides (Yarus 2013). Thus, sporadic polymerization reactions for ribodinucleotides are potentially relevant to the origins of biological systems. We now show experimentally that long tracts of U mediate synthesis of AppA from 5′ AMP via straightforward solution chemistry at moderate temperatures.

## RESULTS

**FIGURE 1.**
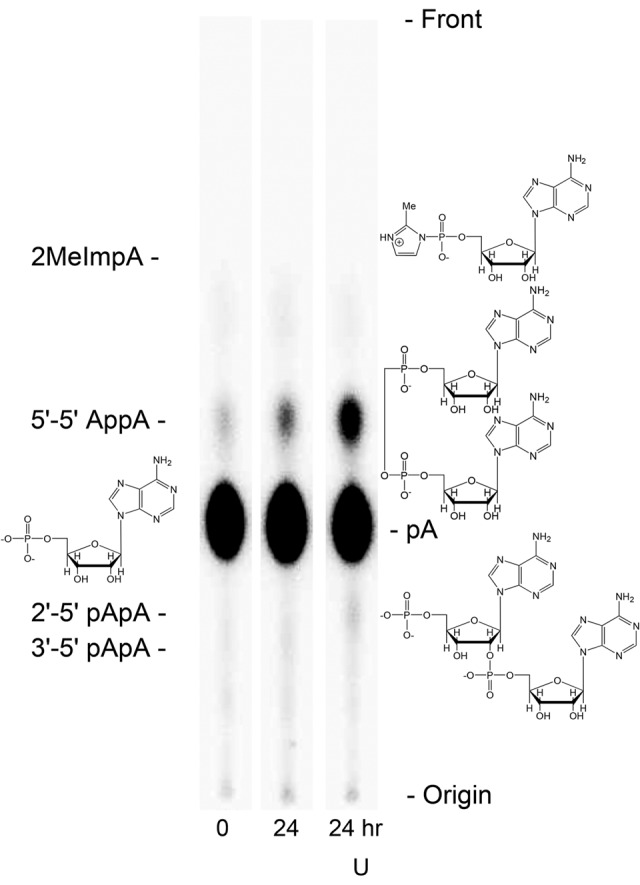
Fractionation of [^32^P]pA radioactivity from three reactions by ion-exchange TLC. On the *left*, “0” means a freshly prepared, unincubated reaction, “24” indicates incubation for 24 h before fractionation, and “24 U” shows the result of 24-h incubation at 10°C with 5 mM poly(U). 2MeImpA (Adenosine 5′phospho-2methylimidazolide) is not radiolabeled and so is invisible after phosphorimaging. Names for some reaction molecules are shown opposite their structures.

We have incubated the 5′ activated adenosine nucleotide 2MeImpA (Adenosine 5′phospho-2methylimidazolide; Fig. 1, right) with [^32^P]pA (5′AMP; Fig. 1, left), in the presence and absence of poly(U). Figure 1 displays a phosphorimage of the routine resolution of reactants and products. Structures for major molecules are shown across from their names alongside the chromatograms. After subjecting a reaction aliquot to ion-exchange TLC, pA incorporated into products like AppA was measured via the fraction of phosphorimaged radioactivity in product spots. Notably, all likely dinucleotide products are resolved, and the predominant product was 5′-5′ AppA throughout these experiments (Fig. 1), sometimes with small amounts of 2′-5′ pApA (just visible, Fig. 1, right). The dinucleotide product, 3′-5′ pApA (which exhibits normal RNA backbone connectivity), is usually not detectable. As shown in the figure, the time scale for reactions at millimolar nucleotide concentrations held at 10°C is hours or days.

**FIGURE 2.**
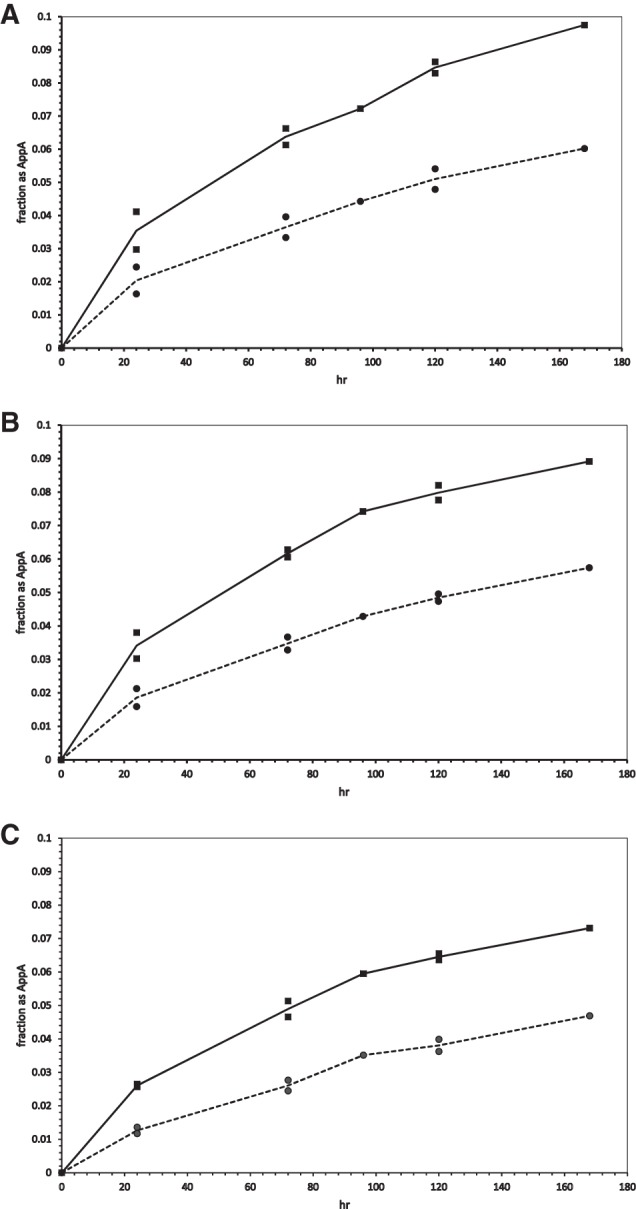
Fraction of total radioactivity in AppA versus time at (*A*) pH 7.00, (*B*) pH 7.55, and (*C*) pH 7.88 at 4°C. Circles and dashed lines mark the chemical reaction; squares and solid lines, the poly(U)-stimulated reaction.

Figure 2A–C shows the fraction of input pA converted into AppA at various times with 4°C reactions held at pH 7.00, pH 7.55, and pH 7.88, ±poly(U). Comparing the panels, poly(U) stimulation is almost independent of pH, though reactions were slightly faster at lower pH. This lack of effect is notable, because 2MeImpA has an imidazolide p*K*_a_ in this range (Kanavarioti et al. 1992), associated with the protonated structure drawn on the right in Figure 1.

**FIGURE 3.**
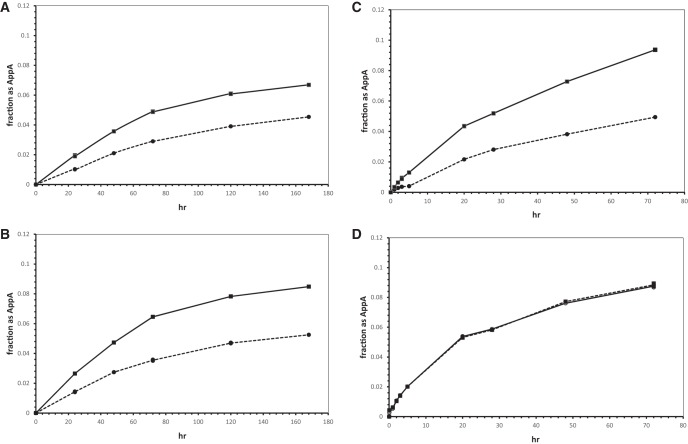
Fraction of total radioactivity in AppA versus time at (*A*) 0°C and pH 7.55, (*B*) 4°C, (*C*) 10°C, and (*D*) 20°C. Circles and dashed lines mark the chemical reaction; squares and solid lines the poly(U)-stimulated reaction.

Figure 3A–D shows unstimulated and poly(U)-stimulated reactions at 0, 4, 10, and 20°C, pH 7.55. Both reactions increase slightly in rate from 0°C to 10°C. In contrast, at 20°C, while the chemical reaction to yield AppA is accelerated, the stimulatory effect of poly(U) reproducibly vanishes. Poly(U) stimulation, but not the reaction itself, uniquely relies on a temperature-dependent transition state whose abundance sharply declines between 10°C and 20°C.

**FIGURE 4.**
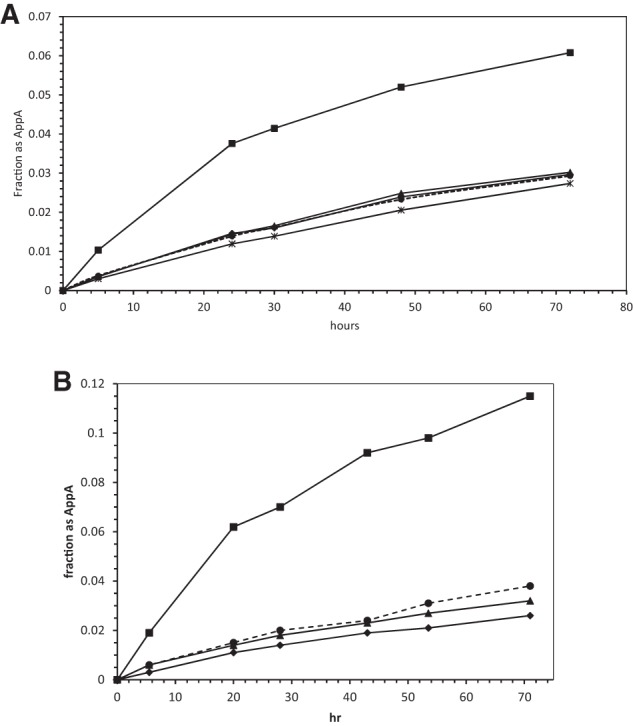
(*A*) Fraction of total radioactivity in AppA at 10°C, with no added polymer (dashed line, small circles), poly(C) (stars), poly(A) (diamonds), poly(G) (triangles), and poly(U) (squares). (*B*) Fraction of total radioactivity in AppA at 10°C, with no addition (dashed line, circles), poly(U) (squares), p(U)_4_ (triangles), and p(U)_8_ (diamonds).

Figure 4A contains data on the specificity of the poly(U) effect. It shows synthesis of AppA with no additions (the background chemical reaction) and with addition of poly(U), poly(C), poly(G), and poly(A). All kinetic points are doubly determined. These data show that AppA synthesis with poly(G) and poly(A) is not distinguishable from the unperturbed chemical reaction. Furthermore, poly(C) addition to 5 mM polymer phosphate slightly inhibits unstimulated production of AppA. In parallel control reactions, stimulation by poly(U) is as usually observed. Thus, AppA production is only increased when a complementary template, capable of normal U:A base pairs, is present. This suggests that the stimulation is mediated by base-pairing between the U's of the poly(U) and the A bases of the 2MeImpA/pA. This can be rationalized by the model below.

Figure 4B further explores the role of poly(U) by measuring the activity of two smaller synthetic oligonucleotides, p(U)_4_ and p(U)_8_, as stimulators of AppA synthesis. In fact, these 3′-5′ oligomers of U do not increase AppA synthesis. It appears that longer tracts of U are required for stimulation of AppA synthesis.

**FIGURE 5.**
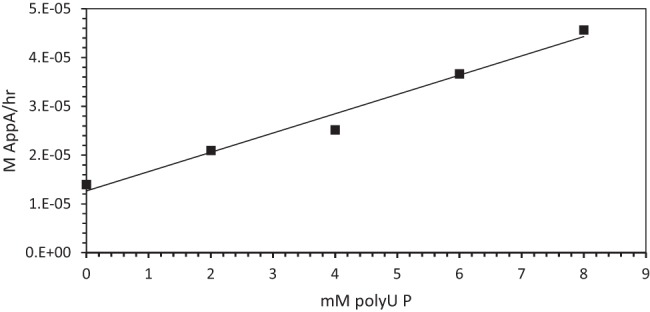
Initial rate of AppA synthesis (M/h) at 4°C versus concentration of poly(U) (in mM nucleotide PO_4_).

Figure 5 explores the role of poly(U) in the transition state for the polymer-stimulated reaction. It shows that the initial rate of AppA synthesis, starting at the basal chemical rate on the left, is stimulated linearly by the addition of poly(U). Thus, it appears that the concentration of active sites for AppA synthesis rises proportionately to added poly(U) concentration. Said another way, there appears to be one molecule of poly(U) participating in each AppA synthesis reaction, rather than a complex of multiple U-containing molecules (which would yield an upwardly curved plot in Fig. 5), which might have seemed possible.

**FIGURE 6.**
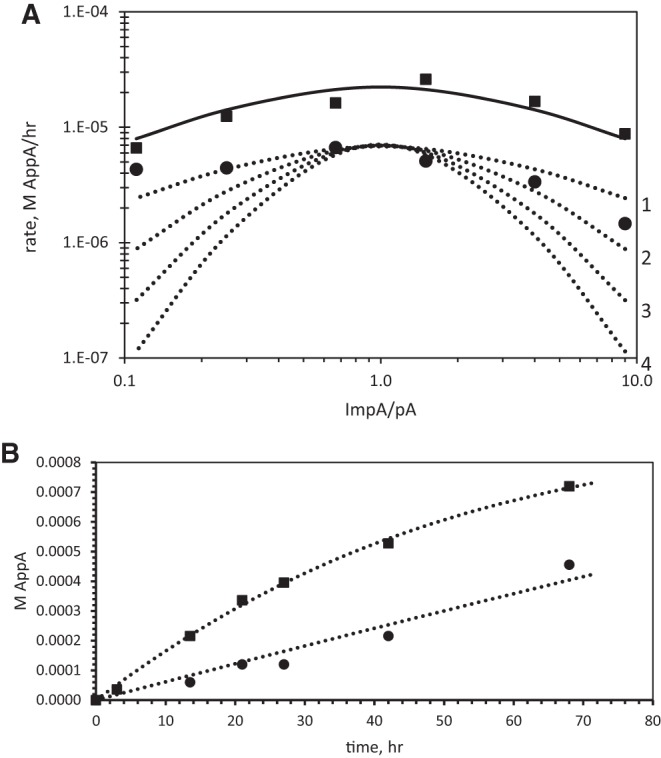
(*A*) Initial rate of AppA synthesis for different substrate ratios at 10°C; plotted versus (2MeImpA)/(pA). Total nucleotide is constant: (2MeImpA) + (pA) = 20 mM. (*Upper* points, solid line) Rate of the reaction with poly(U); (*lower* points, dotted lines) reaction without polymer. (*Lower* dotted curves) Rates of reaction calculated for the lower reaction if rate goes as (2MeImpA) × (pA) (curve labeled 1 at *right*), rate goes as (2MeImpA)^2^ × (pA)^2^ (labeled 2), as (2MeImpA)^3^ × (pA)^3^ (labeled 3), and as (2MeImpA)^4^ × (pA)^4^ (labeled 4 at *right*). (*B*) Example of fit (dotted lines) to data (points) by the mechanism given for simultaneous chemical and poly(U)-catalyzed AppA synthesis, with concurrent hydrolysis of 2MeImpA to pA. Data are for initial concentrations of 12 mM 2MeImpA, 8 mM pA [and 5 mM poly(U) phosphate for the upper data], as shown in Figure 6A.

Figure 6A explores the participation of the nucleotide substrates pA and 2MeImpA in the AppA synthesis reaction, using a logic parallel to that for poly(U) in Figure 5. That is, the ratio of pA/2MeImpA substrates is varied, keeping the total concentration of the two nucleotides constant at 20 mM. We hope to determine that the velocity of AppA synthesis follows the variation of, for example, the product 2MeImpA × pA (as for a second-order reaction between the two nucleotides) or follows 2MeImpA^2^ × pA^2^, or some other simple expression that suggests the nature of the underlying kinetically active complex. The initial rate data, measured over almost 100-fold in initial 2MeImpA/pA in Figure 6A, seem to fit one of the likely expectations.

First, the data for the initial velocity of the poly(U)-stimulated reaction (squares, above in the figure) and the unstimulated chemical reaction (circles below) follow curves with similar shapes. Thus, the stoichiometries of chemical and poly(U)-stimulated AppA synthesis are similar, within experimental precision. The polymeric U, in this sense, enhances a similar reaction that preexists in solution.

Second, both dotted curves have rate maxima near 1:1 ratios of 2MeImpA:pA. This implies that both nucleotide reactants occur in the rate-of-synthesis expression with the same exponent, because the maximum velocity is observed in this plot at the ratio of reactant exponents. For example, there will be a maximum rate at 2MeImpA/pA = 2 if the rate is determined by a rate expression containing (2MeImpA^2^ × pA). Instead, the reaction appears to be controlled by concentrations 2MeImpA × pA or 2MeImpA^2^ × pA^2^, or another expression with equal exponents, which yields maximum velocity when 2MeImpA concentration equals pA.

Third and finally, the AppA synthesis reaction's equal rate-controlling exponents are 1; the reactions are ordinary second-order ones, with respect to the nucleotides. The dotted curves in Figure 6A (lower portion) are calculated for exponents of 1, 2, 3, and 4 (top to bottom; plots labeled on the right). As the order of the reaction increases, the decline away from the central maximum gets steeper. Both the chemical and the poly(U)-stimulated reactions are fit best by the flattest, uppermost dotted arch, characteristic of exponents of 1 (as for a rate ∝ [2MeImpA] × [pA]). Thus, taking these results together with those in Figure 5, we have

$$2\mathrm{MeImpA}+\;\mathrm{pA}\overset{k_{c}}{\to }\mathrm{AppA}+2\mathrm{MeIm}\;(\mathrm{chemical}\;\mathrm{reaction})$$

$$\begin{array}{rl} 2\mathrm{MeImpA}+\mathrm{pA} & +\mathrm{poly}\;U\overset{k_{s}}{\to }\mathrm{AppA}+2\mathrm{MeIm}\\ & +\mathrm{poly}\;U\;(\mathrm{templated}\;\mathrm{reaction}) \end{array}$$

$$\frac{d(\mathrm{AppA})}{dt}=k_{c}\times (2\mathrm{MeImpA})\times (\;\mathrm{pA})+k_{s}\times (\;\mathrm{poly}\;U)\times (2\mathrm{MeImpA})\times (\;\mathrm{pA}),$$

where *k*_c_ and *k*_s_ are, respectively, second- and third-order rate constants for the simultaneous chemical reaction and the poly(U)-stimulated one. The rate constants will be determined below.

To determine realistic rates, it is also necessary to take account of the hydrolysis of 2MeImpA, which decays hydrolytically to the other reactant, pA, on the time scale of these experiments:

$$2\mathrm{MeImpA}\overset{k_{d}}{\to }2\mathrm{MeIm}+\;\mathrm{pA}$$

$$-\frac{d(2\mathrm{MeImpA})}{dt}=\frac{d(\;\mathrm{pA})}{dt}=k_{d}\times (2\mathrm{MeImpA}),$$

where *k*_d_ is the first-order rate of decay. Thus not only does the activated form of A nucleotide decay, but its decay is partially compensated because it increases the concentration of the other nucleotide reactant, pA.

These differential equations were numerically integrated and the rate constants were adjusted to give the best least-squares fit to observed kinetics (see Materials and Methods). These rate constants therefore separate the rates for multiple syntheses alongside simultaneous decay. Figure 6B shows a typical example (12 mM 2MeImpA, 8 mM pA) of integrated least-squares curves (dotted lines) alongside experimental data (points). The mechanism above seems to give a reasonable account of the overall reactions. Figure 6B also exemplifies the reproducible observation of more rapid decay for 2MeImpA in the presence of poly(U); the synthetic rate decays more rapidly with time in the poly(U)-stimulated reaction (upper dotted curve).

The resulting mean rate constants, calculated for the six substrate ratios in Figure 6A, are in Table 1.

**TABLE 1.**
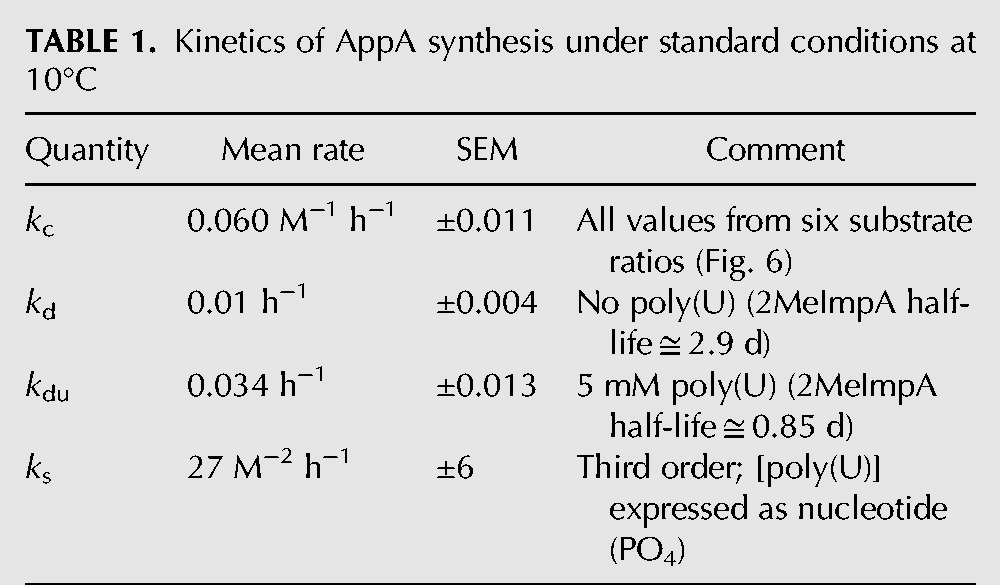
Kinetics of AppA synthesis under standard conditions at 10°C

Addition of poly(U) not only speeds AppA synthesis, but also decay of 2MeImpA (as in Fig. 6B). Enhanced decay of 2MeImpA [faster with 5 mM poly(U)] seems likely to be an example of the previously reported pA catalysis of hydrolysis of imidazolides (Kanavarioti et al. 1992), enhanced by proximity when both nucleotides are paired to a poly(U) template. Thus, poly(U) has dual, partially offsetting effects, stimulating AppA synthesis, but also accelerating destruction of the activated A nucleotide. Finally, derivation of rate constants with acceptable statistics (as in Table 1) from six substrate ratios for the unstimulated (*k*_c_) and six ratios for the poly(U)-stimulated reaction (*k*_s_) is a further argument that the kinetic orders of the chemical and templated reactions (Figs. 5, 6) have been correctly deduced.

**FIGURE 7.**
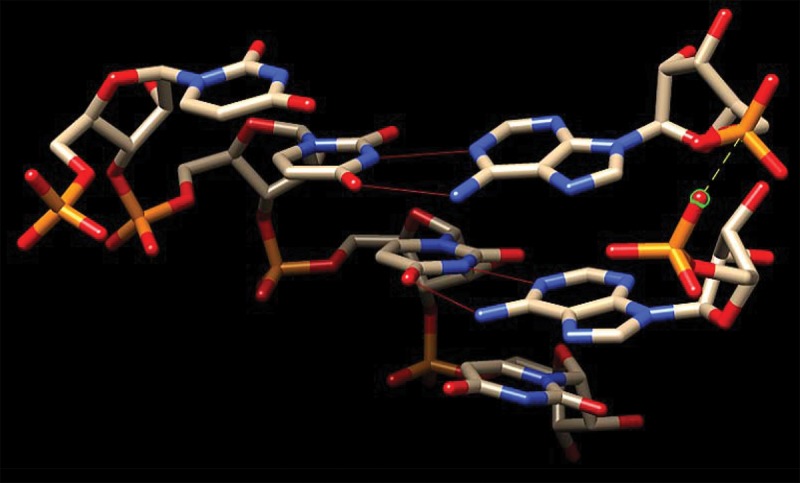
Molecular model for two adjacent helical 5′ pA's paired to a helical, 3′-5′ oligo U tract. p(U)_4_ is shown on the *left*, and two anteparallel pA on the *right*. Thin red lines between A's and U bases mark H-bonds. Approach of oxygen and phosphate atoms in the two pA's is indicated by the dashed yellow line on the *right*.

It might be thought that an unusual nucleotide conformation is required in order to template the formation of a 5′-5′ RNA backbone while paired to a conformationally distinct 3′-5′ RNA backbone. However, this is not necessarily so, as shown by the model in Figure 7. A helical tract of 4 U's, representing the locus of AppA synthesis on poly(U) in the experiments above, is paired to two adjacent pA nucleotides. Ordinary A:U hydrogen bonds are shown in the figure as thin red lines linking paired nucleotide bases.

The two adenine nucleotides are themselves nearly in their normal helical positions and conformations, which were used to build the initial model. In fact, the upper right-hand pA is almost unaltered. Only the lower pA on the right of Figure 7 has been altered (mostly by slightly rotating the glycosidic angle) to bring its phosphate O to 3.5 Å from the phosphate P of the upper pA. The approach of these two atoms is emphasized by a yellow dashed line joining them on the right. Comparing the changed lower right-hand AMP with the relatively unperturbed upper one, it can be readily seen that only small changes have been made to the initial helical conformation to bring potentially reacting atoms together.

Therefore only slight, seemingly probable, changes in conformation are required to displace an activating imidazolide from the upper phosphate and thereby generate the 5′-5′ -O-P-O-P-O- backbone of AppA. These molecular graphics make it quite credible that the formation of one kind of backbone using the other as a template can be observed in solution (e.g., as in Fig. 1), while preserving the base-pairing implied by the observation of template relationships (Fig. 4A). Given the weak interactions expected for two isolated but stacked A:U nucleotide pairs, complete disruption of the reaction between 10°C and 20°C (Fig. 3) is also understandable.

Similarly, for the production of AppA in solution without polymeric assistance, Figure 7 suggests that solution synthesis using spontaneously stacked purines can also be simple, and that plausible stereochemistry (along with elevated reactivities due to the imidazolide) may help to explain the observed predominance of 5′-5′ AppA synthesis (cf. Fig. 1) rather than 2′-5′ or 3′-5′ products. Finally, because these conformations are undemanding and should be universal to paired nucleotides, comparable reactions using other ribonucleotides are likely.

## DISCUSSION

### The reaction

We report here the apparent third-order reaction between poly(U), 5′ activated nucleotide 2MeImpA, and pA (5′ AMP) to yield the dinucleotide A5′pp5′A. The reaction presumably requires at its active center a weak, transient, triple complex containing a poly(U) molecule and two base-paired pA nucleotides, which is disrupted by temperatures of 20°C and above. In fact, the kinetic discussion above is conducted entirely in terms of rates, but preformation of a weak, stacked nucleotide complex in solution, or alternatively, weak but enhanced stacking of A nucleotides when paired to poly(U) both predict rate equations indistinguishable from those written above. Therefore, such stacked complexes are plausible participants in the stimulation of AppA synthesis. Neutral pH and relatively usual monovalent and divalent concentrations are favorable to the formation of this paired transition state (Fig. 7). But surprisingly, in contrast to poly(U), p(U)_4_ and p(U)_8_ oligomers are not effective templates (Fig. 4B); perhaps transient long-flanking stacks of U's are required to stabilize two central stacked, paired, reactive A nucleotides (cf. Fig. 7).

The course of these reactions can be further understood by using the fitted rate constants (at 10°C, as in Table 1). The resolved rates imply that in a normal poly(U) reaction (see Materials and Methods), poly(U)-mediated synthesis is dominant, producing ∼70% of total AppA. When reactions are stopped (Fig. 6B) at 3 d, nearly 93% of the 2MeImpA has been consumed, so productive reaction is almost complete. The ratio 2MeImpA/pA, which was initially 1 (as in Materials and Methods), is now 0.039, because pA has increased from 0.010 M to almost 0.018 M due to imidazolide hydrolysis. Thus, the standard incubation delivers products synthesized at varied 2MeImpA/pA, ranging from one (standard initial conditions) to almost nil (3 d and thereafter). At this stopping point, ∼7% of pA has been converted to 0.7 mM AppA.

### Previous observations

Somewhat related reactions to these have long been recognized. The chemical reaction yielding AppA from pA and ImpA in aqueous solution is well known (Lohrmann and Orgel 1978; Kanavarioti et al. 1992). In addition, pyrophosphate backbones have been observed previously acting as apparent base-paired templates as well as products. Normal 3′-5′ linked oligo C at high concentrations, polymerizing doubly activated ImpdGpIm, accelerates the production of a “copy” with up to 20 dG's linked by pyrophosphates, and poly(U) acts similarly, as an apparent template with doubly activated phosphates (Schwartz and Orgel 1985). Pyrophosphate-linked poly(T) stimulates synthesis of pyrophosphate-linked dA from A dimer substrates (Visscher et al. 1990). Interestingly, large HPLC peaks of uncharacterized smaller products were sometimes observed (Schwartz and Orgel 1985), which may include some produced by the mechanism revealed here.

But most intriguing, a similar reaction to that studied here, poly(U)-templated AppA synthesis using imidazolide activation, was tested at pH 6, 7, and 8 at 0°C and not detected (Weimann et al. 1968), despite concurrent poly(U)-templated synthesis of 2′-5′ pApA in the same reactions. Moreover, easily detected background AppA synthesis was not changed by poly(U) addition when carbodiimide was used for nucleotide activation (Sulston et al. 1968). In addition, it was thought that two strands of poly(U) would be paired to A in the transition state (Sulston et al. 1968), but we likely have only one template poly(U) strand (Fig. 5). There are clear differences between these prior reactions and ours [which include different activating groups, elevated free imidazole, and 10-fold higher poly(U)], but as one result this appears to be the first report of normal RNA-templated synthesis of 5′-5′ coenzyme-like molecules.

### Significance

Small oligonucleotides are capable of complex chemistry. For evolutionary discussion, we are particularly interested in reactions that might yield replication or catalytic activity, as these are required together for evolutionary change in a primordial system of small oligonucleotides (Yarus 2012). In this context, current observations are potentially relevant. First, 3′-5′ linked RNAs are of special interest—these are ubiquitous in present biota, and therefore undoubtedly have a deep evolutionary history. Moreover, 5′-5′ dinucleotides appear of similar antiquity: they resemble widespread modern biomolecules (Yarus 2011), having possible evolutionary continuity with nucleic acid ligation intermediates (Ohtsuka et al. 1976), probably with message caps (Kozak and Shatkin 1978) and likely with enzymatic cofactors for protein enzymes (White 1976). Even more particularly, the frequent appearance of AMP in modern coenzymes, as also in present reactions, was one argument that the 5′-5′ cofactor RNAs were descendants of earlier RNA catalysts (White 1976). Thus, templated but uncatalyzed synthesis of 5′-5′ A dinucleotides suggests an unaccustomed way to specifically produce coenzyme-like molecules (cf. 5′-5′ NAD^+^; Yarus 2011). These observations therefore potentially unite normal RNAs of minimal sequence complexity with a catalytic phenotype (via complementary, chemically active 5′-5′ ribodinucleotides) in an unfamiliar way.

## MATERIALS AND METHODS

### Reactions

Standard reactions are performed at 4°C or 10°C, in 10–15 µL total volume, containing 200 mM HEPES adjusted with KOH, pH 7.55, 50 mM KCl, 50 mM MgCl_2_, 10 mM 2MeImpA, 10 mM pA (5′ AMP) with an added trace of [^32^P]pA (Hartmann Analytic Gmbh, Braunschweig, Germany). When present, other nucleic acids are usually at 5 mM ribonucleotide phosphate. Use of HEPES just above its p*K*_a_ is notable, because it makes reactions particularly resilient to addition of acidic salts and reactants. Similarly, ultra-low retention plasticware, such as RPT tips from USA Scientific Inc., and ultra-low adhesion microtubes from Life Science Products Inc. are very helpful in reproducing reactions containing viscous polymer reactants.

### Nucleotides

2MeImpA (Adenosine 5′-phospho-2-methylimidazolide) was synthesized by the method of Joyce et al. (Joyce et al. 1984a). NppN (e.g., A5′pp5′A) are synthesized as by Kanavarioti et al. (1991), then HPLC-purified, lyophilized, then precipitated with ethanol overnight at −70°C. Finally, nucleotides are dissolved in deionized H_2_O. Ethanol precipitation is an essential step to remove organics introduced by HPLC elution. pNpN are synthesized by Thermo Fisher Scientific Biosciences, and purified by HPLC, lyophilized, then ethanol-precipitated. Polymeric U, A, G, and C were obtained from Sigma-Aldrich as well as Santa Cruz Biotechnology and used with similar results.

### Molecular models

The molecular model shown in Figure 7 was made by forming the 2D hairpin secondary structure of pAAU***UUUU***UAGAAAUUU***AA***UUUU in Assemble2 v1.2 (Jossinet et al. 2010), then homology modeling using known PDB tertiary structures to get a 3D helix. p***UUUU*** from this hairpin, with its central, helically paired p***AA*** were extracted and edited in UCSF Chimera v1.10.1 (Pettersen et al. 2004) to produce the structure shown.

### Chromatography

Microliter volumes of reactions were fractionated on EMD-Millipore thin-layer PEI-cellulose F eluted with 0.5 M LiCl at room temperature (Randerath 1968), then dried and phosphorimaged (BioRad FX). The characteristic positions shown in Figure 1 for 2′-5′, 3′-5′, and 5′-5′ A dinucleotides were identified using standard compounds, nuclease digestions, and mass spectrometry on eluted spots from reactions.

### Analysis of kinetics

Reaction rates were examined using three models.

1. In the first model, preequilibria producing stacked A nucleotides, or equilibrium stacking on poly(U) give rise to a stack containing a catalytically active 2MeImpA-pA apposition.
2. In the second model, no equilibrium is assumed, and each elementary step is integrated, using guessed, but plausible, rates for formation and decay of many possible intermediates neglected in the text, such as the dead end poly(U)-pA-pA complex, or poly(U) paired to AppA product.
3. The third model uses only kinetics; a second-order chemical reaction to yield AppA, a third-order template reaction yielding AppA, and a first-order decay of 2MeImpA are supposed. This is the model emphasized in the text.

All three approaches give indistinguishable numerical results, once differences in notation are allowed for. Because the third model requires the fewest assumptions, using only reactions directly supported by experimental data, these are the equations cited and used for calculations in Results.

The chemical (*k*_c_, second order), template (*k*_s_, third order), and 2MeImpA decay reactions (*k*_d_, first order) were expressed as a system of differential equations (Results), which was numerically integrated using the Rosenbrock integrator of Berkeley Madonna v. 8.3.18 (from Robert Macey and George Oster). The chemical reaction was fit first, and the resulting *k*_c_ was carried over to the subsequent fit of *k*_s_ and *k*_d_ to kinetic data in the presence of poly(U). Values yielding the best least-squares fits to data from all six substrate ratios in Figure 6A were used to get the means and standard errors of the means shown in the Table 1. An example of these computed least-squares fits is shown in Figure 6B.
